# Effect of the Addition of Dried Dandelion Roots (*Taraxacum officinale* F. H. Wigg.) on Wheat Dough and Bread Properties

**DOI:** 10.3390/molecules26247564

**Published:** 2021-12-14

**Authors:** Grażyna Cacak-Pietrzak, Dariusz Dziki, Urszula Gawlik-Dziki, Alicja Sułek, Stanisław Kalisz, Katarzyna Sujka

**Affiliations:** 1 Division of Fruit, Vegetable and Cereal Technology, Department of Food Technology and Assessment, Warsaw University of Life Sciences, 159C Nowoursynowska Street, 02-776 Warsaw, Poland; stanislaw_kalisz@sggw.edu.pl (S.K.); katarzyna_sujka@sggw.edu.pl (K.S.); 2 Department of Thermal Technology and Food Process Engineering, University of Life Sciences in Lublin, Głęboka 31 Street, 20-612 Lublin, Poland; dariusz.dziki@up.lublin.pl; 3 Department of Biochemistry and Food Chemistry, University of Life Sciences in Lublin, 8 Skromna Street, 20-704 Lublin, Poland; 4 Department in Cereal Crop Production, Institute in Soil Science and Plant Cultivation, 8 Czartoryskich Street, 24-100 Puławy, Poland; sulek@iung.pulawy.pl

**Keywords:** *Taraxacum officinale* F. H. Wigg., wheat, bread, baking, physical properties, antioxidants

## Abstract

Dried and crushed dandelion roots (*Taraxacum officinale* F. H. Wigg.) (TO) were used as a formulation additive (at the amount of 0, 1, 3, 4, 5, and 6 g 100 g^−1^ flour) to wheat bread. The farinographic properties of the dough and the physical and chemical properties of the bread were evaluated. It was found that the addition of dried flour caused a significant decrease in water absorption by the flour (1% and higher TO level), an increase in the development time (from 2% to 5% TO addition) and dough stability (3% and 4% TO level), and an increase in dough softening (4% and higher TO level). As the substitution of TO for wheat flour increased, there was a gradual decrease in loaf volume, an increase in specific weight and crumb hardness, and a darkening of the crumb color. The total polyphenol content increased linearly with the percentage increase of dried root additions TO from 0.290 to 0.394 mg GAE g^−1^ d.m_._, which translated into an increase in the antioxidant activity of the bread. It was found that dried crushed roots of *Taraxacum officinale* can be a recipe additive for wheat bread; however, due to their specific smell and bitter aftertaste, the level of this additive should not exceed 3 g 100 g^−1^ flour.

## 1. Introduction

Dandelion (*Taraxacum officinale* F. H. Wigg.) is a perennial plant of the family *Aste-raceae*. There are more than 2500 species of plants belonging to the genus *Taraxacum*, which are morphologically diverse. *Taraxacum officinale* F. H. Wigg. is most likely of European origin and is now widespread in the warm temperate zone [1]. It is a common weed growing in meadows, lawns, and roadside ditches in areas including northern and western Europe, north-western Siberia, Greenland, Iceland, and eastern Canada [2,3]. 

*Taraxacum officinale* F. H. Wigg. is on the list of medicinal plants. The first references to the therapeutic effect of this plant date back to the turn of the 10th and 11th centuries [3]. Both the roots, leaves, and flowers of *Taraxacum officinale* are used in the pharmaceutical industry. The leaves contain mainly sesquiterpene lactones, phenolic acids, and coumarins, as well as vitamin A, the concentration of which is higher than in carrot root [4]. Flavonoids are present in the roots, leaves, and flowers. Large amounts of sesquiterpene lactones are also found in the roots, where coumarins and phenolic acids are also present [3,5]. In addition, the root contains inulin, a polysaccharide with probiotic properties classified as dietary fiber, which is not found in other anatomical parts of this plant [6]. Dandelion roots and leaves are believed to be quite safe, with no side effects or risks of allergic reactions. According to the Food Drug Administration, dandelion is generally recognized as a safe food additive [7]. 

*Taraxacum officinale* F. H. Wigg. exhibit antioxidant, antibacterial, anti-inflammatory, anticancer, hypolipidemic, and hepatoprotective properties [1,3,6,7,8,9]. The antioxidant properties are due to the presence of many bioactive metabolites belonging to different groups, mainly phenolic compounds [10,11], as well as flavonoids, terpenoids, glycosides, reducing sugars, and tannins [8,11]. Aqueous and ethanolic extracts of the leaves and roots of this plant are effective against pathogenic microorganisms, including *Escherichia coli* and *Staphylococcus aureus* [12,13], whereas the root extracts show greater bactericidal properties against *S. aureus* [13]. Leaf and root extracts can be used in dentistry as a disinfectant that strongly inhibits the growth of *Enterococcus faecalis* [14]. In vivo studies on guinea pigs have shown very good efficacy of *Taraxacum officinale* leaf extract in the treatment of tracheal inflammation [15], similarly to in vivo mouse studies on acetic acid-induced angiogenesis [16]. It was also shown that the leaf extracts have a protective effect on lung tissue in the course of acute lung injury [17] and on liver tissue in chronic liver failure [9,18]. Hepatoprotective effects and a reduction of renal dysfunction were also obtained after treatment with *Taraxacum officinale* root extracts during in vivo studies in rats [11]. Leaf extracts are highly effective in inhibiting invasion of breast cancer cells and root extracts in inhibiting invasion of prostate cancer cells [19] and pancreatic cancer [20]. In addition, leaf extracts have immunosuppressive effects and can be used in chemotherapy as a natural immunostimulant [21]. González-Castejón et al. [22] proved that extracts from dandelion leaves and roots partially inhibit the process of fat cell formation in the human body. *Taraxacum officinale* roots, thanks to their inulin content, influence the reduction of the total cholesterol concentration with a simultaneous increase in the HDL fraction and reduce the level of triglycerides in blood and liver cells [23,24]. An infusion of dandelion flowers is used to alleviate acne symptoms and is also used for throat, bronchial, and rhinitis ailments. Syrup made from the flowers is used to alleviate coughs, as well as during urinary tract infections, detoxification of the body, and as an aid in the early stages of atherosclerosis treatment. The milky juice squeezed directly from the stalk is a cure for warts. The young leaves can be eaten raw, in a salad, or as a cocktail [1,3]. 

In recent years, there has been a trend towards using natural substances contained in plants as a source of antioxidants and functional ingredients in foods. 

Currently, the use of *Taraxacum officinale* roots in the food industry is quite limited. The roots of this plant are the raw material from which inulin is extracted and used in the microbiological production of fructose syrup [25]. The roots are the raw material for teas with toxin-cleansing properties and, when roasted, are used as a coffee substitute [26]. Kenny et al. [27] demonstrated that *Taraxacum officinale* root extracts can be used as a potential natural preservative to retard oxidative processes in food products. Kęska et al. [28] used the combination of ultrasound with dandelion extracts to prepare a functional beef-meat product with enhanced antioxidant potential. 

So far, there have been no studies on the possibility of using the roots of *Taraxacum officinale* as a recipe additive for bread. Therefore, the aim of this study was to evaluate the effect of the addition of dried crumbled roots of this plant on the baking properties of flour and the quality of wheat bread and to determine the level of this addition acceptable by consumers. 

## 2. Results and Discussion

### 2.1. Physico-Chemical Properties of Breadmaking Raw Materials

Wheat flour used as the basic raw material for bread baking was characterized by an appropriate baking value, containing 12.76 ± 0.02% d.m. of total protein and 30.7 ± 0.05% wet gluten of optimal quality (Index Gluten 75 ± 2), and the activity of amylolytic enzymes was at an average level (falling number 281 ± 1 s). The flour moisture content was 13.8 ± 0.0%, while the total ash content (0.71 ± 0.0% d.m.) was in accordance with the flour type declared by the producer. Dried *Taraxacum officinale* root had a slightly lower moisture content than wheat flour (12.2 ± 0.01%) and a more than five times higher content of total ash (3.83 ± 0.05% d.m.). Moreover, according to the information included in a producer label, it contained 72% carbohydrates, 7.2% proteins, and 0.7% unsaturated fatty acids. 

### 2.2. Water Absorption of Mixtures and Rheological Characteristics of Dough Enriched with Taraxacum officinale 

Substitution of wheat flour with dried crushed roots of *Taraxacum officinale* caused changes in the farinograph water absorption of the mixtures. Water absorption decreased from 57.5% (control sample) to 55.5% (sample with 6% TO) (Table 1). Statistically significant differences compared to the control sample were found already at 1% substitution of wheat flour with TO. The roots of TO are rich in inulin [29]. Salinas et al. [30] found that inulin causes a decrease in the water absorption of wheat flour. 

The literature data on the influence of various additives of plant origin on water absorption of wheat flour are not unequivocal. Dziki et al. [31] found that the addition of dried crushed sumac fruit (*Rhus coriaria*), similar to the TO addition in this study, resulted in a reduction of water absorption from 57.7% (control sample) to 54.2% (5% addition). In other studies, wheat flour substitution with purified fibers of different origin [32], dried minced onion waste [33], dried leaves of Moldavian dragonhead (*Dracocephalum moldavica*) [34], and dried leaves of rockrose (*Cistus incanus)* [35], increased the amount of absorbed water. In our study, substitution of wheat flour with TO in amounts from 2% to 5% caused a significant increase in the dough development time (5.0, 5.5, 5.6, and 5.3 min, respectively) in comparison with the control sample (4.7 min). A significant increase of the dough stability time in comparison to the control was found in the samples with 3% and 4% TO addition, while 6% TO addition caused a significant decrease of this parameter. At 4% and higher levels of this addition, a significant increase of dough softening occurred. Similar trends have been reported by other authors studying the properties of wheat dough with the addition of fiber-rich raw materials [32,33,36]. The weakening of the dough structure may be explained by interactions between fiber and gluten, which may impede protein hydration [32,37]. In addition, the partial replacement of wheat flour with other plant raw materials, containing no gluten proteins but rich in fiber, results in a less cohesive and weaker dough structure due to the lower amount of gluten proteins [36]. Literature [6] shows that the dried roots of *Taraxacum officinale* used in the study are rich in inulin, a polysaccharide indigestible in the human digestive tract and classified as dietary fiber. The mentioned authors determined the content of this component in the roots of TO at the level of 436.29 mg g^−1^. 

### 2.3. Yield, Volume, and Density of Bread Crumb Enriched with Taraxacum officinale

The substitution of wheat flour with dried TO roots resulted in a decrease in bread yield, from 147.3% (control sample) to 144.5% (sample with 6% TO), which was due to a decrease in water absorption by the mixtures with dried TO roots, but these changes were not statistically significant (Table 2). A volume reduction when chia seeds (*Salvia hispanica)* were added to the wheat bread recipe was obtained by Romankiewicz et al. [38]. However, no significant effect of microencapsulated onion peel extracts on wheat bread yield was shown by Czaja et al. [39]. In other studies, bread yield increased when dried crushed leaves of Moldavian dragonhead were added to the recipe (*Dracocephalum moldavica)* [34] and leaves of rockrose (*Cistus incanus*) [35]. 

The volume of bread decreased with increasing the share of dried crushed roots of *Taraxacum officinale* and the specific mass of crumb increased gradually with increasing the share of this additive from 1% to 6% (Table 2). A significantly smaller loaf volume compared to the control sample was noted for bread with 3% and higher TO share, and a significantly higher crumb specific mass, for bread with a 5% and 6% TO share. A similar trend was observed after the addition to the recipe of wheat bread of dried crushed leaves of the Moldavian dragonhead *(Dracocephalum moldavica)* [34], leaves of rockrose (*Cistus incanus)* [35], leaves of nettle (*Urtica dioica)* [40], dried ground berries of sumac (*Rhus co-riaria)* [31], powdered onion waste [33], turmeric *(Curcuma longa*) [41], and chia seeds (*Salvia hispanica*) [38]. Czaja et al. [39] and Pasrija et al. [42] found no significant effect of the addition of microencapsulated onion peel extracts and green tea leaf extracts on bread yield, but this could be due to the relatively low proportion of these additives in the bread recipe. The decrease in volume of wheat bread enriched with plant raw materials, shown in many studies, can be explained by the presence of fiber in them, which interacts with proteins and weakens the gluten network of the dough, resulting in a reduction in the amount of CO_2_ retained in the dough during fermentation and, consequently, a smaller volume of the loaf [38]. The increase in crumb density shown in our study was a consequence of the reduction in volume of the loaf, which led to greater crumb compacting. 

### 2.4. Textural Characteristics of Bread Enriched with Taraxacum officinale

Substitution of wheat flour with dried TO roots caused slight changes in the textural properties of the bread crumb. The springiness, springiness, and cohesiveness of bread crumb with TO addition were comparable to the control sample (Table 3). The exception was crumb hardness, which increased linearly from 7.1 (control sample) to 12.1 N (sample with 6% TO). The increase in the crumb hardness of wheat bread was also caused by the addition to its recipe of leaves of dried rockrose (*Cistus incanus*) [35], dried leaves of Moldavian dragonhead (*Dracocephalum moldavica*) [34], berries of sumac (*Rhus coraria*) [31], dried leaves of nettle (*Urlica dioica*) [40], turmeric (*Curcuma longa*) [41], micronized extracts of green tea leaves [42], and artichoke (*Cynara scolymus*) [43]. The demonstrated increase in the crumb hardness of wheat bread enriched with plant raw materials may be explained by the presence of fiber and polyphenols. According to Pasrija et al. [42], these substances may compete for water with starch granules and cause higher crumb hardness. Our studies did not show any influence of TO used as a recipe additive in wheat bread on the springiness, resilience, and cohesiveness of the bread crumb. 

### 2.5. Crumb Color of Bread Enriched with Taraxacum officinale

**Figure 1:**
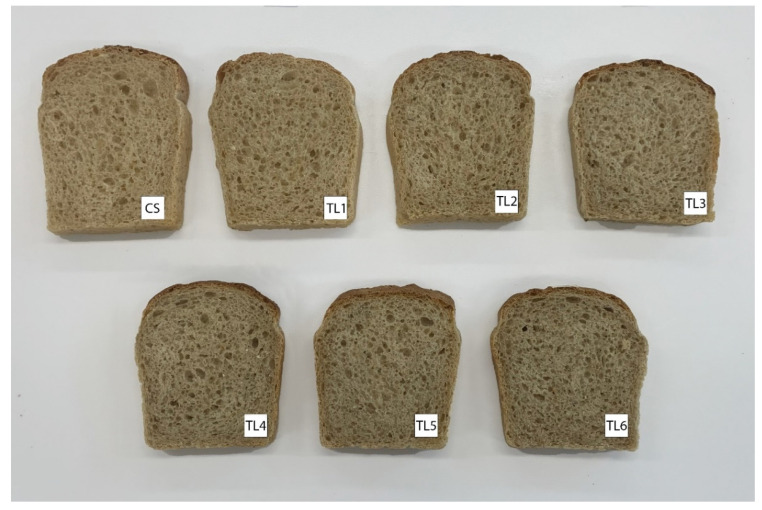
Appearance of wheat bread with different amounts of *Taraxacum officinale*.

Substitution of wheat flour with dried *Taraxacum officinale* roots had an effect on the bread crumb color. With the increase of the percentage of this additive, the brightness parameter (L*) gradually decreased, indicating a darkening of the crumb color; the parameter a* increased, and the parameter b* decreased, indicating an increase in the percentage of red color and a decrease in the percentage of yellow color (Table 4). The biggest differences in crumb color were observed between the control sample and the sample with 6% TO addition (Figure 1). To illustrate the variation in the bread crumb color, the total difference in color was calculated (ΔE). According to Romankiewicz et al. [38], the International Commission on Illumination classifies the overall color difference as follows: ΔE in the range 0–2, a difference is unrecognizable; the range between 2.0 and 3.5 indicates that the color difference is recognizable by an inexperienced observer; and at values above 3.5, the color difference is distinct. In our study, ΔE between the crumb of the control bread and bread with added TO ranged from 3.39 (1% TO share) to 7.37 (6% TO share). It means that the color of the bread crumb with TO added at the level of 2% and above significantly differed from the color of the bread crumb of the control bread and it should be noticed even at the level of 1% of TO addition. Significant changes in bread crumb color after adding various natural additives to the recipe are indicated by the results of many studies [31,34,35,38,39,40,41,42,43]. Różyło et al. [44] showed that plant additives can be used as natural food colorings, which are more acceptable to consumers than the synthetic or semi-synthetic colorings now commonly used in the food industry. 

### 2.6. Polyphenol Content and Antioxidant Properties of Bread Enriched with Taraxacum officinale

Previous studies have demonstrated that root extracts from *Taraxacum officinale* have both in vivo and in vitro antioxidant activity [45]. Substitution of wheat flour with dried crushed roots of *Taraxacum officinale* increased the total polyphenol (TPC) content of bread from 0.290 (control sample) to 0.394 mg GAE g^−1^ d.m. (sample with 6% TO) (Table 5). This was due to the almost seven times higher content of these compounds in the dried TO root, compared to wheat flour (respectively: 2.145 ± 0.01 and 0.313 ± 0.00 mg GAE g^−1^ d.m.). A higher polyphenol content translated into a statistically significant reduction in the value of EC_50_, indicating an increase in the antioxidant activity of the bread. The increase in the antioxidant activity of the bread was confirmed in both tests conducted. In the test with the cation radical ABTS^•+^, the value of EC_50_ decreased from 1348.9 (control sample) to 362.4 mg d.m. mL^−1^ (sample with 6% TO), while in the test with the radical DPPH, the value of EC_50_ decreased from 255.4 (control sample) to 182.9 mg d.m. mL^−1^ (sample with 6% TO). The main phenolic acid contained in wheat flour is ferulic acid [46]. The roots of TO contain mainly chicoric acid, as well as phenolic acids, i.e., caffeic acid, chlorogenic acid, ρ-coumaric acid, ferulic acid, ρ-hydroxybenzoic acid, protocatechuic acid, vanillic acid, and syringic acid coumarins (umbelliferone, esculetin, scopoletin, cichoriin, and aesculin) [47]. Besides phenolic compounds, the antioxidant activity of TO roots is also shaped by various flavonoids, such as luteolin, isorhamnetin, apigenin, and quercetin derivates (rutinosides and pentoside) [5]. Lis et al. [48] found TO roots are a valuable source of secondary metabolites, such as hydroxyphenylacetate inositol esters, with antioxidant, antiplatelet, and anticoagulant properties.

### 2.7. Sensory Evaluation of Bread Enriched with Taraxacum officinale

**Figure 2:**
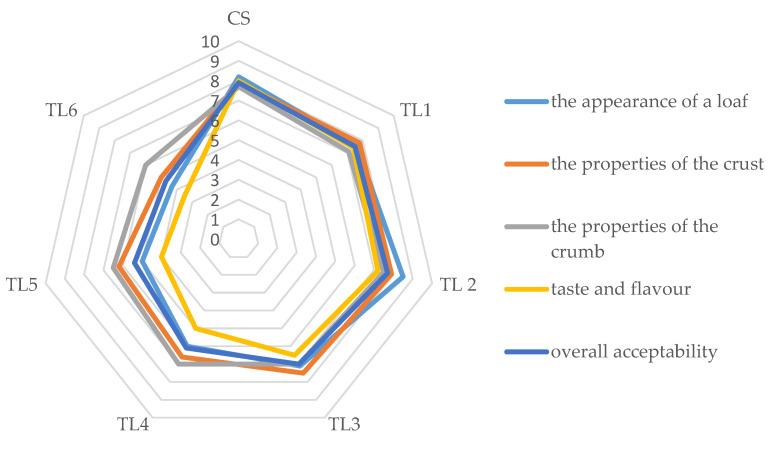
Results of sensory evaluation of bread enriched with *Taraxacum officinale.* CS—control sample of bread, TL1, TL2, TL3, Tl4, TL5, TL6—bread with 1, 2, 3, 4, 5, and 6 g 100 g^−1^ of *Taraxacum officinale*, respectively.

Sensory evaluation showed that the substitution of wheat flour with TO influenced its sensory properties, which translated into overall consumer acceptance (Figure 2). Breads with 1%, 2%, and 3% TO addition were given comparable scores for loaf appearance with the control sample. The bread with 6% TO was rated lowest, which was mainly due to the poor rise of the loaf. In terms of crust characteristics, the highest marks were given to the control sample and breads with up to 3% TO, while breads with a higher proportion of TO lost smoothness of the crust surface and showed too dark a color. The sensory evaluation panel generally had no major complaints about the crumb characteristics. The crumb of all bread samples was adequately soft and elastic and showed no tendency to crumble, but according to some evaluators, the color of the crumb of the bread with 6% TO was too dark. The biggest differences compared to the control sample were in taste and smell. At 4% and higher TO addition, an unusual smell and a distinct bitter aftertaste were perceptible. This was due to the presence of sesquiterpene lactones, bitter-tasting substances found in TO [4]. High consumer acceptance, comparable to the control sample, was achieved by breads with 2%, 1%, and 3% TO, and with a higher proportion of this additive, the sensory evaluation results of the bread were much lower. The results of several studies [31,33,34,35,39,40,41] indicate that it is often necessary to reduce the level of substitution of wheat flour with natural additives, due to the deterioration of the sensory characteristics of bread. For example, the proportion of chia (*Salvia hispanica*) seeds acceptable to consumers in wheat bread was determined at 6% [38], turmeric (*Curcuma longa*) at 4% [41], while dried leaves of nettle (*Urlica dioica*) at 2% [40]. A maximum of 3% should be the additive of dried leaves of the Moldavian dragonhead (*Dracocephalum moldavica*) [34], dried leaves of rockrose (*Cistus incanus*) [35], berries of sumac (*Rhus coraria*) [31], and dried onion waste [33]. In view of the pronounced bitterness, the maximum level of microencapsulated onion skin extracts should not exceed a level of only 0.5% [39].

## 3. Materials and Methods

### 3.1. Raw Material

The raw materials used to make the bread dough were commercial bread wheat flour type 750 (Polskie Młyny, Warsaw, Poland), dried roots of *Taraxacum officinale* F. H. Wigg. (Flos, Mokrsko, Poland), compressed yeast (Lallemand Sp. z o.o., Józefów, Poland), and table salt (Cenos Sp. z o.o., Września, Poland). Dried dandelion roots were ground into particles (size less than 0.3 mm) in a laboratory impact mill WŻ-1 (Research Institute of the Baking Industry, Bydgoszcz, Poland).

### 3.2. Chemical Composition of Raw Material

In wheat flour, the total protein content was determined by the Kjeldahl method (N∙5.70) on a Kjeltec 8200 (Foss, Hilleroed, Denmark) according to AACC Method 46-11.02 [49], wet gluten content and Index Gluten by the mechanical method on a Glutomatic 2200 (Perten Instruments, Stockholm, Sweden) AACC Method 38-12.02 [49], and falling number by the Hagberg–Perten method in the Falling Number 1400 (Perten Instrument, Stockholm, Sweden) AACC Method 56-81B [49]. In wheat flour and dried *Taraxacum officinale* (TO) roots, total ash content was determined by ashing in a muffle furnace FCF S (Czylok, Jastrzębie Zdrój, Poland) at 900 °C for 60 min according to the AACC Method 08-01.01 [49] and the moisture content by drying according to the AACC Method 44-40.01 [49] in a SUP-65 convection dryer (Wamed, Warsaw, Poland).

### 3.3. Water Absorption and Rheological Characteristics of Dough

To evaluate the water absorption and rheological characteristics of the dough, mixtures were prepared in which wheat flour was replaced by dried *Taraxacum officinale* roots at 1%, 2%, 3%, 4%, 5%, and 6% by the weight of the flour. The control sample was wheat flour. The determination was carried out using a Farinograph-E model 810114 with a mixer for 50 g of flour (Brabender Gmbh & Co. KG, Duisburg, Germany) according to the AACC Method 54-21.02 [49]. Water absorption was read from a burette and the rheological properties of the doughs (development time, solidification time, softening) were determined from a normal curve plot using the Farinograph v.5 computer program.

### 3.4. Baking Procedure of Bread

Bread for this study was prepared under laboratory conditions from dough prepared using the direct method described by Romankiewicz et al. [38]. The basic dough recipe consisted of wheat flour (100%), water, compressed yeast (3%), and table salt (1.5%). Wheat flour was replaced with TO at 1%, 2%, 3%, 4%, 5%, and 6% by the weight of the flour. Water was added in the amount needed to obtain a dough of 350 FU consistency, and the water addition was determined by the farinograph water absorption results. All dough recipe ingredients were mixed for 3 min in a SP 800A SPAR mixer (Food Machinery, Taichung, Taiwan). The doughs were fermented in a fermentation chamber for 90 min (breakthrough after 60 min) at 30 °C. The doughs were then divided into 250 g pieces and, after being placed in molds, subjected to a final rise for 45 min at 30 °C. Baking took place in a DC-32E electric oven (Sveba Dahlen, Fristad, Sweden) for 30 min at 230 °C. After baking, the breads were cooled and packed in polyethylene bags and stored for 24 h at 20 °C.

### 3.5. Yield, Volume, and Density of Bread Crumb

After 24 h of baking, the bread yield was calculated, the loaves were weighed, and their volume and crumb-specific weight were determined. The volume of the loaf was determined using a 3D scanner (NextEngine, West Los Angeles, CA, USA) and calculated using a computer program (MeshlLab, ISTI-CNR Research Centre, Rome, Italy) and then converted into 100 g of bread [38]. The density of the bread crumb was determined from the mass of a sample of known volume [50].

### 3.6. Textural Properties of Bread Crumbs

The textural properties of the bread crumb were determined using a TA.XT2i type texture analyzer (Stable Microsystem, Surrey, UK) according to the methodology given by Armero and Collar [51]. Cylindrical samples (22 mm diameter) cut from 20 mm thick slices of bread were compressed using a head equipped with a 25 mm diameter mandrel, and the mandrel travel speed was 1 mm s^−1^. A 40% sample penetration with a 45 s interval between the first and second compression was applied. Hardness, springiness, resilience, and cohesiveness were determined from the obtained TPA (Profile Texture Analysis) curve.

### 3.7. Color Measurements

The color parameters of the raw materials (wheat flour, dried *Taraxacum officinale* roots) and bread were determined using the CIE-L*a*b* reflectance method, where L* denotes the color brightness, a* red/green saturation, and b* yellow/blue saturation. The measurement was performed with a CR-200 colorimeter (Konica Minolta, Osaka, Japan). From the mean values of the L*a*b* color components, the absolute difference in color (ΔE) was calculated between the bread made from wheat flour (control sample) and bread made with dried dandelion root [44].

### 3.8. Total Phenolic Content and Antioxidant Activity

#### 3.8.1. Preparation of Samples

The content of total polyphenolic compounds and antioxidant activity were determined in raw materials (flour, dried *Taraxacum officinale* roots) and in crushed dried bread samples. For extraction, samples of 1 g were weighed, 25 cm^3^ of PBS buffer (pH = 7.4) was added and kept for 60 min at room temperature. The resulting homogenate was centrifuged, and supernatants were separated. The extraction was performed three times. The supernatants were combined and stored in a dark place at −20 °C [52]. 

#### 3.8.2. Total Polyphenols Content

The total polyphenol content was determined by the Folin–Ciocalteau spectrophotometric method according to the methodology given by Singleton and Rossi [53]. From each sample, 0.5 mL of buffer extract was taken, added with 0.5 mL of distilled water, 2 mL of Folin’s reagent (1:5 H_2_O), and 2 mL of 10% Na_2_CO_3_ solution, all mixed thoroughly and left for 60 min. The absorbance of the solution was measured using a Mini 1240 UV spectrophotometer (Shimadzu, Kyoto, Japan) at 720 nm against a blank (PBS buffer). 

The total phenolic contents were expressed as mg gallic acid equivalents (mg GAE) per g of dry mass (dm). All treatments were carried out in triplicate. The results were calculated using the standard calibration curve of gallic acid (based on 10 measurement points from 0 to 1 mg∙ml^−1^, R^2^ = 0.995).

#### 3.8.3. Ability to Quench DPPH Free Radicals

The free radical quenching capacity was determined spectrophotometrically according to the method given by Brand-Williams et al. [54]. In total, 0.25 mL of DPPH solution in ethanol and 0.01 mL of buffer extract were measured into a tube, shaken, and allowed to stand for 15 min. Color was measured using a microplate spectrophotometer (BioTek, Santa Clara, CA, USA) at 725 nm. The ability to quench DPPH free radicals was expressed as EC_50_.

#### 3.8.4. Quenching Capacity of ABTS^•+^ Cation Radicals 

The quenching capacity of ABTS^•+^ cation radicals was determined spectrophotometrically according to the methodology given by Re et al. [55]. In total, 1.8 mL of ABTS^•+^ radical and 0.04 mL of buffer extract were measured into a tube. After 5 min, color measurement was carried out using a Mini 1240 UV spectrophotometer (Shimadzu, Kyoto, Japan) at 734 nm. The quenching ability of ABTS^•+^ cation radicals was expressed as EC_50_.

### 3.9. Sensory Evaluation of Bread

Sensory evaluation of the bread was carried out by a panel of 55 panelists aged 23–58 years, 24 h after baking. The bread samples were evaluated on a 9-point hedonic scale, where 1 meant “very undesirable”, 5 “neither desirable nor undesirable”, and 9 “very desirable”. The evaluation took into account the following bread quality characteristics: loaf appearance, crust appearance, crumb characteristics, taste and aroma, and overall acceptability [41].

### 3.10. Statistical Analysis

All determinations were performed in a minimum of three replicates. Statistical ana-lysis of the results obtained was performed using Statistica 13.3 software (TIBCO Software, Palo Alto, CA, USA). Analysis of variance (ANOVA) was performed, and homogeneous groups were determined using the Tukey test. Calculations were performed assuming a significance level of α = 0.05.

## 4. Conclusions

The substitution of part of wheat flour with dried crumbled roots of *Taraxacum officinale* caused lower water absorption of the mixtures and weakening of the dough structure. As a result, the productivity and volume of bread decreased. The addition of TO had a beneficial effect on the color of the bread crumb, and increased the content of polyphenols and the antioxidant activity of the bread measured by the ability to quench DPPH free radicals and ABTS^•+^. cation radicals. On the basis of the obtained results, it was concluded that enrichment of the bread recipe with dried TO roots in an amount not exceeding 3% of 100 g^−1^ wheat flour allows functional bread with increased antioxidant activity and acceptable sensory qualities to be obtained.

## Figures and Tables

**Table 1:** Farinograph indices for wheat flour and wheat flour with *Taraxacum officinale*.

Sample	Water Absorption (%)	Development Time (min)	Stability of Dough (min)	Degree of Softening (FU)
CS	57.5 ± 0.10 ^a^	4.7 ± 0.10 ^c^	7.3 ± 0.26 ^b^	70.0 ± 1.00 ^c^
TL 1	56.7 ± 0.06 ^b^	4.9 ± 0.06 ^bc^	7.5 ± 0.20 ^b^	71.0 ± 1.73 ^c^
TL 2	56.5 ± 0.06 ^b^	5.0 ± 0.06 ^b^	7.7 ± 0.17 ^b^	69.0 ± 1.00 ^c^
TL 3	56.3 ± 0.12 ^c^	5.5 ± 0.20 ^a^	8.3 ± 0.10 ^a^	70.0 ± 2.65 ^c^
TL 4	56.0 ± 0.10 ^cd^	5.6 ± 0.20 ^a^	8.3 ± 0.30 ^a^	78.0 ± 2.65 ^b^
TL 5	56.0 ± 0.06 ^cd^	5.3 ± 0.20 ^ab^	7.7 ± 0.20 ^b^	80.3 ± 1.53 ^ab^
TL 6	55.5 ± 0.06 ^e^	4.9 ± 0.06 ^bc^	6.5 ± 0.20 ^c^	85.3 ± 2.31 ^a^

CS—control sample of wheat flour, TL 1, TL 2, TL 3, Tl 4, TL 5, TL 6—wheat flour with 1, 2, 3, 4, 5, and 6 g 100 g^−1^ of *Taraxacum officinale*, respectively. Note: Means with a different letter (^a–e^) in the same column are significantly different (α = 0.05).

**Table 2:** Yield, volume, and density of bread crumb enriched with *Taraxacum officinale*.

Sample	Yield of Bread (%)	Volume of Bread (cm^3^ 100 g^−1^)	Density of Crumb (g cm^3 (−1)^)
CS	147.3 ± 1.57 ^a^	366.0 ± 14.66 ^a^	0.32 ^b^ ± 0.01 ^b^
TL 1	146.3 ± 2.34 ^a^	353.2 ± 3.84 ^a^	0.33 ^b^ ± 0.02 ^b^
TL 2	145.3 ± 0.61 ^a^	350.5 ± 4.27 ^a^	0.33 ^b^ ± 0.01 ^b^
TL 3	145.3 ± 1.04 ^a^	331.1 ± 4.26 ^b^	0.35 ^b^ ± 0.00 ^b^
TL 4	145.2 ± 0,89 ^a^	323.8 ± 1.31 ^b^	0.35 ^b^ ± 0.00 ^b^
TL 5	145.1 ± 1.21 ^a^	292.2 ± 3.26 ^c^	0.38 ^a^ ± 0.01 ^a^
TL 6	144.5 ± 0.36 ^a^	287.6 ± 2.09 ^c^	0.39 ^a^ ± 0.01 ^a^

CS—control sample of bread, TL 1, TL 2, TL 3, Tl 4, TL 5, TL 6—bread with 1, 2, 3, 4, 5, and 6 g 100 g^−1^ of *Taraxacum officinale*, respectively. Note: Means with a different letter (^a–c^) in the same column are significantly different (α = 0.05).

**Table 3:** Textural characteristics of bread enriched with *Taraxacum officinale*.

Sample	Hardness (N)	Resilience (mm)	Springiness (mm)	Cohesiveness (-)
CS	7.1 ± 0.30 ^d^	0.31 ± 0.01 ^a^	0.94 ± 0.01 ^a^	0.62 ± 0.01 ^a^
TL 1	8.7 ± 0.26 ^c^	0.29 ± 0.01 ^a^	0.93 ± 0.01 ^a^	0.61 ± 0.01 ^a^
TL 2	9.2 ± 0.26 ^bc^	0.29 ± 0.01 ^a^	0.92 ± 0.01 ^a^	0.60 ± 0.01 ^a^
TL 3	9.4 ± 0.35 ^bc^	0.30 ± 0.00 ^a^	0.92 ± 0.01 ^a^	0.61 ± 0.01 ^a^
TL 4	9.9 ± 0.10 ^b^	0.30 ± 0.00 ^a^	0.91 ± 0.01 ^a^	0.61 ± 0.01 ^a^
TL 5	11.2 ± 0.44 ^a^	0.30 ± 0.01 ^a^	0.92 ± 0.01 ^a^	0.62 ± 0.00 ^a^
TL 6	12.1 ± 0.46 ^a^	0.28 ± 0.02 ^a^	0.91 ± 0.01 ^a^	0.61 ± 0.01 ^a^

CS—control sample of bread, TL 1, TL 2, TL 3, Tl 4, TL 5, TL 6—bread with 1, 2, 3, 4, 5, and 6 g 100 g^−1^ of *Taraxacum officinale*, respectively. Note: Means with different letter (^a–d^) in the same column are significantly different (α = 0.05).

**Table 4:** Crumb color parameters of bread enriched with *Taraxacum officinale*.

Sample	L* (-)	a* (-)	b* (-)	ΔE (-)
CS	65.08 ± 0.14 ^a^	1.14 ± 0.03 ^e^	14.90 ± 0.04 ^a^	-
TL 1	61.76 ± 0.21 ^b^	1.71 ± 0.01 ^d^	14.48 ± 0.04 ^b^	3.39
TL 2	61.44 ± 0.08 ^b^	2.20 ± 0.04 ^c^	14.35 ± 0.03 ^b^	3.83
TL 3	60.90 ± 0.19 ^c^	2.26 ± 0.03 ^bc^	14.11 ± 0.10 ^c^	4.40
TL 4	59.62 ± 0.14 ^d^	2.36 ^b^ ± 0.06 ^b^	13.94 ± 0.04 ^cd^	5.68
TL 5	59.56 ± 0.04 ^d^	2.65 ^a^ ± 0.06 ^a^	13.78 ± 0.11 ^d^	5.83
TL 6	58.10 ± 0.11 ^e^	2.68 ^a^ ± 0.02 ^a^	13.09 ± 0.10 ^e^	7.37

CS—control sample of bread, TL 1, TL 2, TL 3, Tl 4, TL 5, TL 6—bread with 1, 2, 3, 4, 5, and 6 g 100 g^−1^ of *Taraxacum officinale*, respectively. Note: Means with a different letter (a–e) in the same column are significantly different (α = 0.05). CS—control sample of bread, TL1, TL2, TL3, Tl4, TL5, TL6—bread with 1, 2, 3, 4, 5, and 6 g 100 g^−1^ of *Taraxacum officinale*, respectively.

**Table 5:** Total phenolic content and antioxidant activity of bread incorporated with *Taraxacum offi-cinale*.

Sample	TPC	DPPH	ABTS^•+^
	mg GAE g^−1^ d.m_._	EC_50_ (mg d.m. mL^−1^)	
CS	0.290± 0.001 ^c^	255.4 ± 13.26 ^a^	1348.9 ± 79.50 ^a^
TL 1	0.319± 0.018 ^c^	225.1 ± 12.81 ^b^	1318.2 ± 69.65 ^a^
TL 2	0.356± 0.012 ^b^	221.6 ± 11.52 ^bc^	613.3 ± 35.42 ^b^
TL 3	0.361 ± 0.009 ^ab^	210.7 ± 10.67 ^bcd^	510.9 ± 28.37 ^bc^
TL 4	0.362 ± 0.040 ^ab^	193.2 ± 9.29 ^cd^	507.3 ± 27.11 ^bc^
TL 5	0.369 ± 0.111 ^ab^	188.1 ± 7.20 ^d^	442.9 ± 20.22 ^cd^
TL 6	0.394 ± 0.108 ^a^	182.9 ± 5.18 ^d^	362.4 ± 19.34 ^d^

CS—control sample of bread, TL 1, TL 2, TL 3, Tl 4, TL 5, TL 6—bread with 1, 2, 3, 4, 5, and 6 g 100 g^−1^ of *Tara-xacum officinale*, respectively. Note: Means with a different letter (^a–d^) in the same column are significantly different (α = 0.05).

## Data Availability

The data presented in this study are available on request from the corresponding author.

## Sample Availability

Samples of the compounds are not available from the authors.
